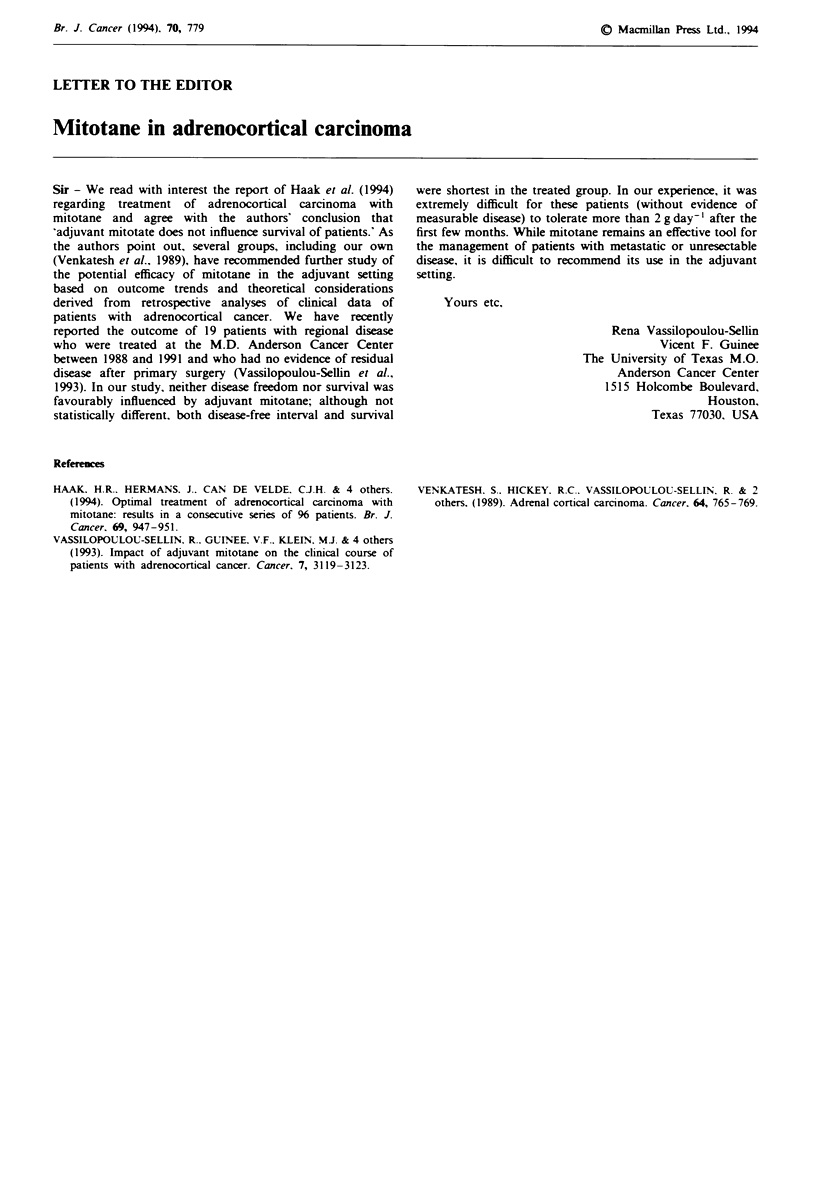# Mitotane in adrenocortical carcinoma.

**DOI:** 10.1038/bjc.1994.397

**Published:** 1994-10

**Authors:** R. Vassilopoulou-Sellin, V. F. Guinee


Br. J. Cancer (1994), 70, 779 © Macmillan Press Ltd., 1994

LETTER TO THE EDITOR

### Mitotane in adrenocortical carcinoma

**Sir** – We read with interest the report of Haak *et al.* (1994) regarding treatment of adrenocortical carcinoma with mitotane and agree with the authors' conclusion that 'adjuvant mitotate does not influence survival of patients.' As the authors point out, several groups, including our own (Venkatesh *et al.*, 1989), have recommended further study of the potential efficacy of mitotane in the adjuvant setting based on outcome trends and theoretical considerations derived from retrospective analyses of clinical data of patients with adrenocortical cancer. We have recently reported the outcome of 19 patients with regional disease who were treated at the M.D. Anderson Cancer Center between 1988 and 1991 and who had no evidence of residual disease after primary surgery (Vassilopoulou-Sellin *et al.*, 1993). In our study, neither disease freedom nor survival was favourably influenced by adjuvant mitotane; although not statistically different, both disease-free interval and survival were shortest in the treated group. In our experience, it was extremely difficult for these patients (without evidence of measurable disease) to tolerate more than 2 g $day^{-1}$ after the first few months. While mitotane remains an effective tool for the management of patients with metastatic or unresectable disease, it is difficult to recommend its use in the adjuvant setting.

Yours etc,

Rena Vassilopoulou-Sellin
Vicent F. Guinee
The University of Texas M.O.
Anderson Cancer Center
1515 Holcombe Boulevard,
Houston,
Texas 77030, USA

**References**

HAAK, H.R., HERMANS, J., CAN DE VELDE, C.J.H. & 4 others. (1994). Optimal treatment of adrenocortical carcinoma with mitotane: results in a consecutive series of 96 patients. *Br. J. Cancer*, **69**, 947–951.

VASSILOPOULOU-SELLIN, R., GUINEE, V.F., KLEIN, M.J. & 4 others (1993). Impact of adjuvant mitotane on the clinical course of patients with adrenocortical cancer. *Cancer*, **7**, 3119–3123.

VENKATESH, S., HICKEY, R.C., VASSILOPOULOU-SELLIN, R. & 2 others, (1989). Adrenal cortical carcinoma. *Cancer*, **64**, 765–769.